# Complete mitochondrial genome of the top hat blenny, *Omobranchus fasciolatoceps* Richardson (Blenniiformes: Blenniinae) in Southern China Coast

**DOI:** 10.1080/23802359.2022.2116948

**Published:** 2022-09-02

**Authors:** Tao Zhang, Shiwei Wu, Lei Yang, Hongling Ping, Bin Lu, Jun Liang, Xuejun Yu, Yanming Sui, Yimeng Liu, Huilai Shi

**Affiliations:** a Zhejiang Province Key Lab of Mariculture and Enhancement, Marine Fisheries Research Institute of Zhejiang, Zhoushan, China; b School of Marine and Biological Engineering, Yancheng Institute of Technology, Yancheng, China; c Feixian Maternal and Child Health Care Hospital, Liyin, China; d Key Laboratory of Sustainable Utilization of Technology Research for Fisheries Resources of Zhejiang Province, Marine Fisheries Research Institute of Zhejiang, Zhoushan, China; e College of Life Science, Linyi university, China; f Chinese Academy of Fishery Sciences East China Sea Fishery Research Institute Department, Aquaculture Technology Laboratory, Shanghai, China

**Keywords:** Mitochondrial genome, *Omobranchus fasciolatoceps*, phylogenetic analysis

## Abstract

The complete mitochondrial genome sequence of *Omobranchus fasciolatoceps* was firstly described in this article. The total length of mitogenome was 16,569 bp. It contains 13 protein-coding genes, 22 tRNA genes, and two ribosomal RNA genes. The overall base composition of H-strand was 29.04% A, 27.14% C, 27.89% T, and 15.93% G, with an A＋T bias of 56.93%. The phylogenetic analysis results showed that the *O. fasciolatoceps* was most closely related to *O. elegans*.

*Omobranchus fasciolatoceps* (Richardson 1846) belongs to genus *Omobranchus* in family Blenniidae of Blenniiformes (Kawaguchi et al. 1999). It is a native species distributed along southern China coast, and mainly appears in estuaries and the ocean shallows near shore. It commonly grows to an average length of 8 cm and can adapt to a wide range of temperatures. Its fleshy crest and zebra-striped face make this substrate dweller appealing.

The complete mitochondrial genome of *O. fasciolatoceps* was firstly reported in this paper. From that, we expected to provide useful information on the population genetics of *O. fasciolatoceps* and promote further molecular phylogenetic studies. The sample of *O. fasciolatoceps* in this article was collected from the south China coast (122°1′20″ E, 29°2′33″ N). The specimen was deposited at the Key Laboratory of Fisher Equipment and Engineering, Ministry of Agriculture (contacts, Liu Yimeng, liuymeng@foxmail.com), with the voucher number Ofacsi20190710001. DNA libraries were prepared using Nextera DNA Flex Library Prep (Illumina, San Diego, CA, USA). High-throughput sequencing was performed on an Illumina HiSeq X-10 sequencer in 150-bp paired-end mode. Subsequently, the clean paired-end reads were assembled by the Perl script Novoplasty v 4.3.1(Dierckxsens et al. 2017).

The whole length of *O. fasciolatoceps* mitogenome was 16,569 bp. The nucleotide composition of the heavy strand was 29.04% A, 27.14% C, 27.89% T, and 15.93% G, with an A＋T bias of 56.93%. It contained 13 protein-coding genes (PCGs), 22 tRNAs and 2 rRNAs. Most genes were located on the heavy strand, except for *ND6* and 8 tRNA genes (*tRNA^Gln^*, *RNA^Ala^*, *tRNA^Asn^*, *tRNA^Cys^*,*tRNA^Tyr^*, *tRNA^Ser^*, *tRNA^Glu^*, *tRNA^Pro^*). The control region (913 bp) was identified between *tRNA^Pro^* and *tRNA^Phe^*. Eleven PCGs shared the start codon ATG, except for COXI and ND6 (GTG as start codon). Six PCGs (ND1, COX1, ATPase 8, ND4L, ND6) were terminated with the typical stop codons (TAA), while the incomplete termination codons (T––/TA–) were identified among seven PCGs (ND2, COX2, ATPase 6, COX3, ND3, ND4, and Cyt b). In addition, ND5 ended with AGA.

For phylogenetic analysis, 15 taxa were selected: 11 species from order Blenniiformes (including Omobranchus fasciolatoceps), two species from order Cichliformes, and two species from order Lutjaniformes. Nucleotide sequences of the other mitochondrial genomes were downloaded from GenBank. The individual nucleotide sequences of 13 PCGs, two rRNAs genes, and 22 tRNAs genes were aligned by the ClustalW program (Larkin et al. 2007). A Maximum Likelihood phylogenetic tree (shown in Figure 1) was constructed using MEGA 6 (Stamatakis 2006; Tamura et al. 2013) with GTR + I + G model. It showed *O. fasciolatoceps* was situated near *O. elegans*, and had a closer relationship with *Petroscirtes breviceps*.

**Figure 1.**
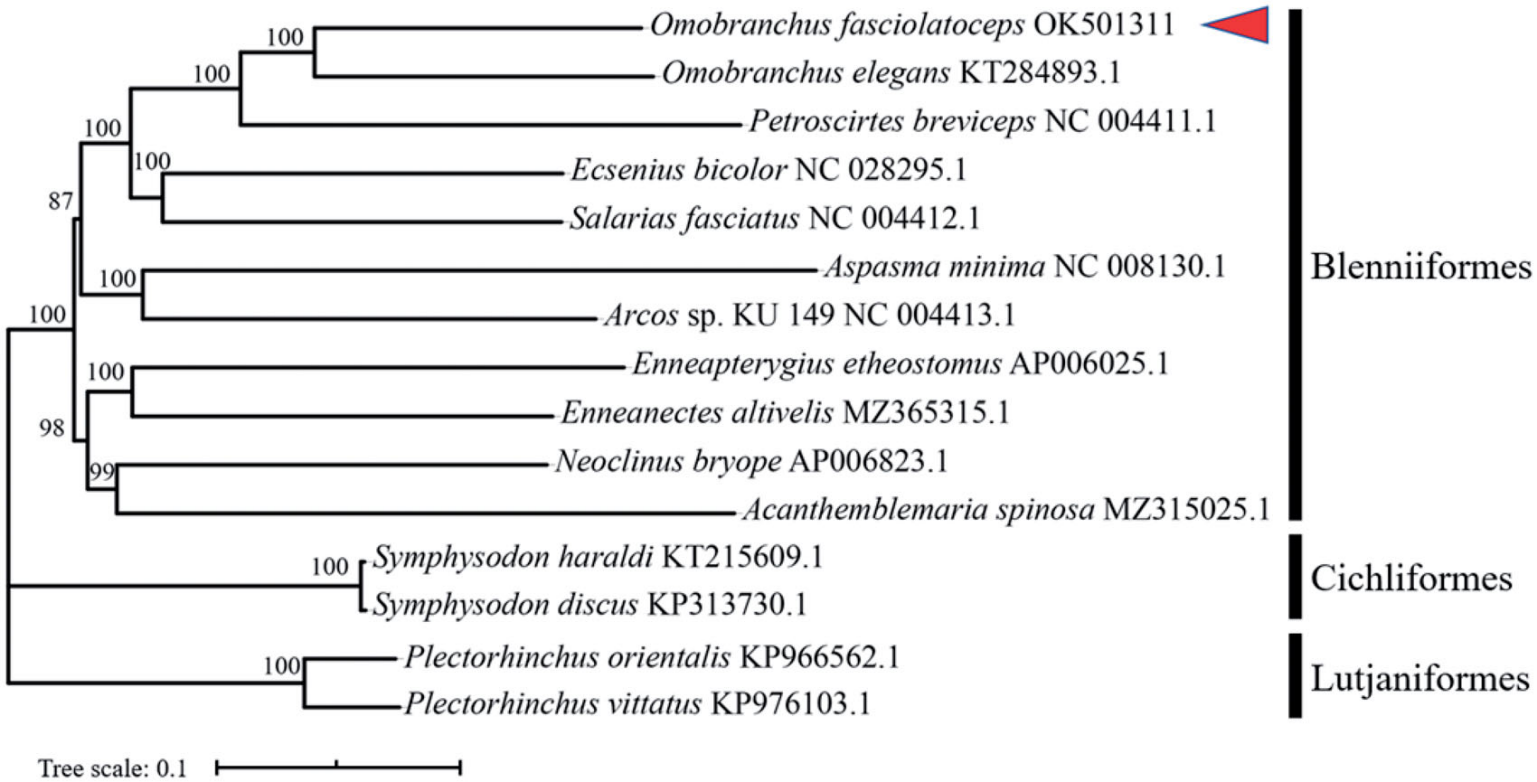
A phylogenetic tree for *Omobranchus fasciolatoceps* and other 14 species representing orders Blenniiformes, Cichliformes, and Lutjaniformes based on assembled nucleotide sequences of 13 protein-coding, two rRNAs genes, and 22 tRNAs genes. The number on each node indicates the values of ultrafast bootstrap (UFB) of 1000 replications.

## Data Availability

The genome sequence data that support the findings of this study are openly available in GenBank of NCBI at [https://www.ncbi.nlm.nih.gov] under the accession no. OK501311. The associated **BioProject**, **SRA**, and **Bio-Sample** numbers are PRJNA770635, SRR16298008, and SAMN22225431 respectively.
